# Between a Rock and a Hard Place: Considering “Freebirth” During Covid-19

**DOI:** 10.3389/fgwh.2021.603744

**Published:** 2021-02-18

**Authors:** Mari Greenfield, Sophie Payne-Gifford, Gemma McKenzie

**Affiliations:** ^1^King's College London, London, United Kingdom; ^2^University of Hertfordshire, Hatfield, United Kingdom

**Keywords:** freebirth, pregnancy, choice, COVID-19, maternity, childbirth, LGBTQ+

## Abstract

**Background:** The global coronavirus (Covid-19) pandemic concerns all people, but has a specific effect on those who are expecting a baby during this time. The advice in the UK changed rapidly, with 14 different sets of national guidance issued within 1 month. Individual NHS Trusts released various guidance relating to the withdrawal of homebirth services, the closure of birth centers, restrictions on the number of birth partners (if any) allowed during labor, and whether any visitors were allowed to attend after birth. With the landscape of maternity care changing so rapidly, research was carried out to provide real-time data to capture the lived experiences of expectant families.

**Methods:** A mixed methods online survey was carried out over 2 weeks between 10th and 24th April 2020. The survey was open to those in the third trimester of pregnancy, those who had given birth since the beginning of the “lockdown” period in the UK, and the partners of pregnant women and people who were in these circumstances. The survey asked questions about how respondents' holistic antenatal experiences had been affected, whether their plans for birth had changed, and the effect of these changes on respondents' emotional wellbeing. Of the 1,700 responses received, 72 mentioned that they had seriously considered “freebirthing” (giving birth without a healthcare professional present).

**Findings:** An analysis of the respondents' reasons for considering freebirth was conducted, finding that reasons for considering freebirth were complex and multifaceted. Lesbian, bisexual, pansexual, and queer women were more likely to have considered freebirth than heterosexual people (*p* < 0.001).

**Conclusions:** Considering giving birth without a healthcare professional present is unusual in the Global North and represents an emerging field of study. The literature examining the reasons that people consider freebirth shows a variety of underlying motivations. A global pandemic represents a new factor in such considerations. The findings from this research can help inform maternity service planning in future crises.

## Introduction

The global coronavirus (Covid-19) pandemic concerns all people but has a specific effect on those who are expecting a baby during this time. Perinatal care, like emergency medical care, is time-sensitive, and cannot be delayed and then accessed later. In the first days of the lockdown in the UK, rapid response research was planned to understand the real-time social and cultural impact on the lived experience of people accessing maternity care in the UK. Our research question was:

What are the experiences of perinatal care of those who are due to have a baby in the first months of lockdown in the UK, and how do they feel about these experiences?

Our article is drawn from this wider research project, which used an online survey of parents. The survey comprised of three main elements: capture of demographic information; a psychometric tool that was administered to those who had given birth; and a series of open-ended questions. The survey opened on 10th April 2020 and closed on 24th April 2020.

One of the themes that emerged from the open-ended questions was that 72 respondents had given serious consideration to freebirthing. This paper specifically discusses the experiences of those respondents, examining both why they considered this option, and their feelings about freebirth.

### Freebirth

Freebirth occurs when someone:

“intentionally giv[es] birth without health care professionals (HCPs) present in countries where there are medical facilities available to assist them (1).”

Although legal in the UK, freebirth is typically viewed as a non-mainstream and stigmatized birthing decision. The subject is under-researched and there is a paucity of academic literature on the phenomenon. Existing studies are largely qualitative and focus on the motivations of women in Western nations such as USA (2), UK (3), Ireland (4), Canada (5), Australia (6), Norway (7), and The Netherlands (8).

Such studies highlight that women decide to freebirth for a range of reasons. These include a previous traumatic birth (6), dissatisfaction with the care offered by perinatal services (7), and an inherent belief in the undisturbed physiological processes of birth (3). An inability to access care based on “logistics” and geographical distance to a maternity unit (9) and limitations on homebirths have also played a role in women's decision-making (4).

### Freebirth and the Covid-19 Pandemic

In the first weeks of lockdown in the UK, the advice for expectant parents changed rapidly. On the 9th March 2020 the Royal College of Obstetricians and Gynecologists (RCOG) issued guidance suggesting pregnant women were not at greater risk from coronavirus that the general population. However, a week later, the UK Government guidance stated pregnant women were one of the most vulnerable groups. Within a few days, RCOG advised NHS Trusts to consider closing smaller maternity units (10).

Despite the proven safety of out of hospital settings for low-risk births (11, 12), in the first days of lockdown individual NHS Trusts released different guidance relating to the withdrawal of homebirth services, and the closure of birth centers and midwife-led units (MLUs). Restrictions were also placed on the number of birth partners—if any—allowed during labor, and whether any visitors (and who they were) were allowed to visit after birth.

The uncertainty and confusion around this advice meant that pregnant people became concerned as to how these restrictions would impact their rights and experiences during labor and birth. As a result, national human rights charities such as the Association for Improvements in the Maternity Services (AIMS) and Birthrights, published a range of literature to support people impacted by these restrictions [e.g., (13, 14)]. Further, it became apparent to midwives that some women were contemplating removing themselves entirely from NHS perinatal care and freebirthing their babies. Concerned by this, on 30th April 2020 the Royal College of Midwives (RCM) issued a clinical guidance note for midwives advising on how to support women intending to freebirth (15).

Quantitative data about freebirth is almost non-existent. It is unknown, for example, how many people per year freebirth their babies in the UK. Demographics relating to freebirthers' socio-economic background, ethnicity, age, and parity do not exist. In short, within the UK context, there has never been a quantitative study undertaken that attempts to collect such data. Given this lack of statistical data relating to freebirth, the rates of increased interest in freebirthing due to the COVID-19 pandemic remain unclear. However, communities such as the Freebirth and Emergency Childbirth Support Group—a UK fee-based Facebook group—have been created on social media during the pandemic. This group provided information to almost 300 expectant parents, healthcare professionals and birth supporters. The emergence of groups such as this during lockdown suggests a genuine interest from a range of people in learning more about freebirth.

## Methods

### Data Collection

An online survey was undertaken to capture the experiences of those in the UK who had given birth, or were due to give birth, between the 9th March 2020 and the 3rd July 2020, or whose partners had given birth or were due to give birth between these dates. The dates chosen ensured participants had either recently become parents or were in the third trimester of pregnancy at the time of the research. The survey collected demographic data, used a psychometric tool to measure support in labor and birth, and included a large number of open-ended questions about respondents' experiences.

Participants were asked to indicate whether they or their partner was pregnant, their baby's date of birth or due date and their local healthcare service trust. Participants were also asked to indicate their ethnicity, age, disability, sexual orientation, and gender. The main part of the survey consisted of free text boxes which asked when participants became aware of Covid-19, and when they understood that it might impact their pregnancy and birth plans. It also asked about their plans for birth and whether they had changed, whether they were accessing private healthcare providers, whether other elements of perinatal care had changed, and how they felt about becoming a parent during a pandemic. A psychometric scale for those who had given birth was also included, but the results are not discussed in detail here. All questions after the consent and birth/due date were optional. The questionnaire tool is attached at Supplementary Table 1.

The survey was promoted and carried out entirely online due to the practicalities of the pandemic, and also to allow as many people to respond as possible. An advert with a hyperlink to the survey was shared on Twitter from both the first author's personal account and a King's College account. On Facebook, the advert was shared in generic birth groups, “due in” groups, homebirth groups, cesarean birth groups, parenting groups and locality-based birth groups. Two human rights charities, Birthrights and the Association for Improvements in Maternity Services (AIMS) were involved in helping design the survey, and in promoting it through their online social media. The questionnaire was open from 10th to 24th April 2020, and 1,754 responses were received.

### Case Selection

This article reports in detail on the responses that related to freebirth. The psychometric scale data was removed, and a textual search of the full responses was carried out in the Excel spreadsheet for the terms:

“Freebirth”
“Unattended”
“Unassisted”
“Free [AND] birth”

The last search term produced a high number of false positive results such as “stress free birth,” so all results for this search were manually checked before being included. The word “alone” was searched for (in the spreadsheet) but returned too many vague results. The mention of fear related to giving birth alone may refer to freebirthing, but is more likely to refer to giving birth without a partner, a situation many respondents were unhappy with.

Responses which included these terms were then read in full by the lead researcher (MG), and included in the freebirth dataset if they indicated that the participant or their partner had considered freebirth at any point, or if they had had a freebirth. This resulted in responses from 72 people who had considered or had a freebirth being included in the dataset. The full responses (excluding the psychometric scale) from these participants were then uploaded into NVivo. Two responses which mentioned freebirth were excluded from the analysis as these responses mentioned that the participants were too scared to consider freebirth, or that they were concerned other women might choose to freebirth. A second check of the full database was conducted by the second researcher (SPG) to ensure that all cases had been correctly identified.

### Analysis

The demographic data from the full dataset were compiled so as to compare with those considering freebirth. The dataset of 72 responses was then thematically analyzed using NVivo. Thematic analysis is a methodology often used within qualitative research in the social sciences, because it can generate rich detail from the data, whilst also providing an overall organizational structure to compare and discuss the data within. It is used for “identifying, analyzing and reporting patterns (themes) within data” [(16), p. 79].

As the aim of this research was to capture the real-time lived experiences of expectant parents during lockdown, we wanted to employ an analytical methodology that would provide a rich description of the dataset rather than a theoretically driven methodology.

Six stages of analytic process are described by Braun and Clarke (16) as part of a robust thematic analysis process. These are: familiarization, initial coding, searching for themes, reviewing themes, naming and describing themes, and producing a report. Reading and re-reading the responses which mentioned freebirth to determine whether they should be included in the analysis provided the necessary familiarization for the researchers. The dataset was then transferred to NVivo, and the lead researcher used an inductive approach to generate initial codes from the open-ended questions. This initial coding was organized into themes, providing a map of the data, which were reviewed by the second researcher (SPG).

Each theme was then named and described, drawing on the data to ensure that participants' voices remained the center of the analysis. The themes are presented above in Table 1, and a full codebook of the themes is available at Supplementary Table 1. The three main themes are: where birth was planned to happen before the pandemic; what non-NHS support respondents considered; and respondents' reasons for considering freebirth.

**Table 1:** Themes identified.

**Theme**	**Subtheme**
Planned place of birth	
Non-NHS support available/considered	Doula
	Independent midwife (IM)
Reasons for considering freebirth	Avoid hospital
	Previous traumatic birth
	Coercion
	Birth partner potentially excluded
	Uncertainty
	Access to water
	Childcare
	Distance/access to transport
	Timing

The findings above use the themes identified to form the structure of this article. Simple quantitative analysis was also undertaken with the freebirth dataset, firstly to produce descriptive statistics of the demographics of the participants, but also to turn qualitative answers into quantitative ones by turning open-ended answers into closed ones. Turning qualitative data into quantitative data can be one of the purposes of qualitative research (17).

## Results

### Quantitative Findings

This section begins by identifying the demographic characteristics of the participants who had considered freebirth. We then go on to examine participants' plans for birth before the pandemic.

Of the 72 participants who said they had seriously considered freebirth during the pregnancy, 69 were women who were pregnant at the time of the research. Two participants were women who had given birth since the 9th March, and one participant was a man whose partner was pregnant. This division in the types of participant is roughly in line with the total dataset, where 1,385 were still pregnant at the time of the research, 336 had given birth, and 33 were the partner of someone who was pregnant or had given birth.

**Figure 1:**
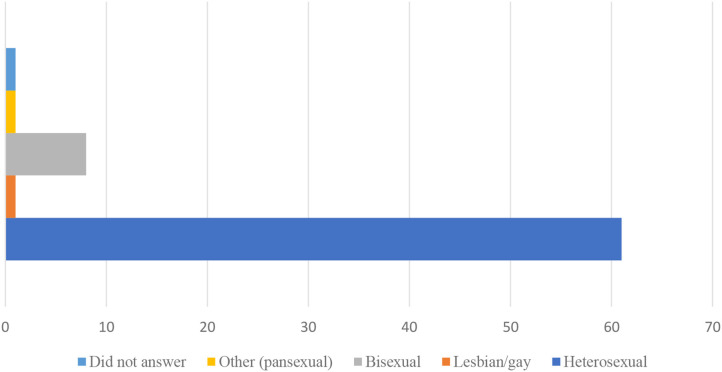
Sexual orientation of participants considering freebirth.

**Figure 2:**
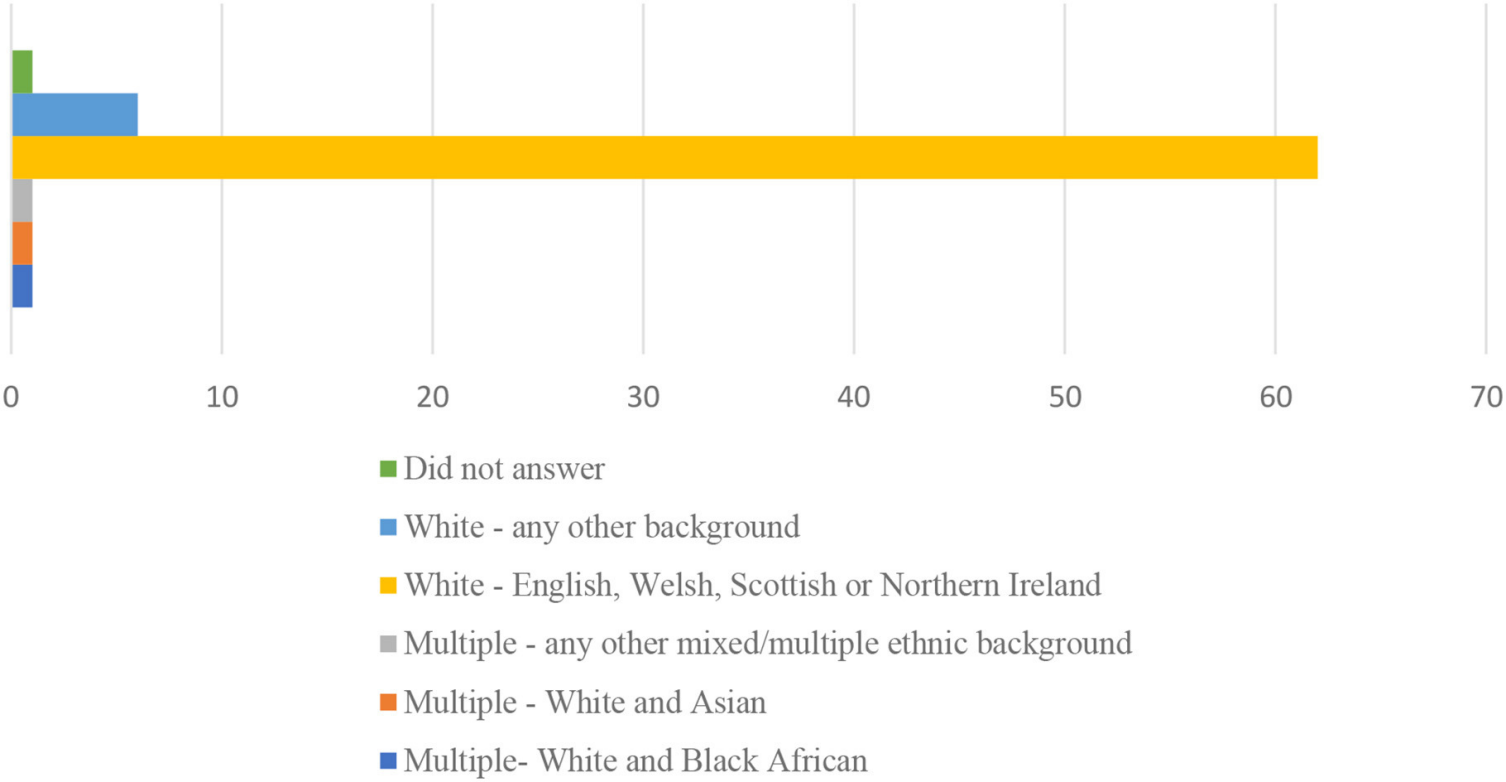
Ethnicity of participants considering freebirth.

The majority of participants were white, heterosexual women, as is shown in Figures 1, 2.

**Figure 3:**
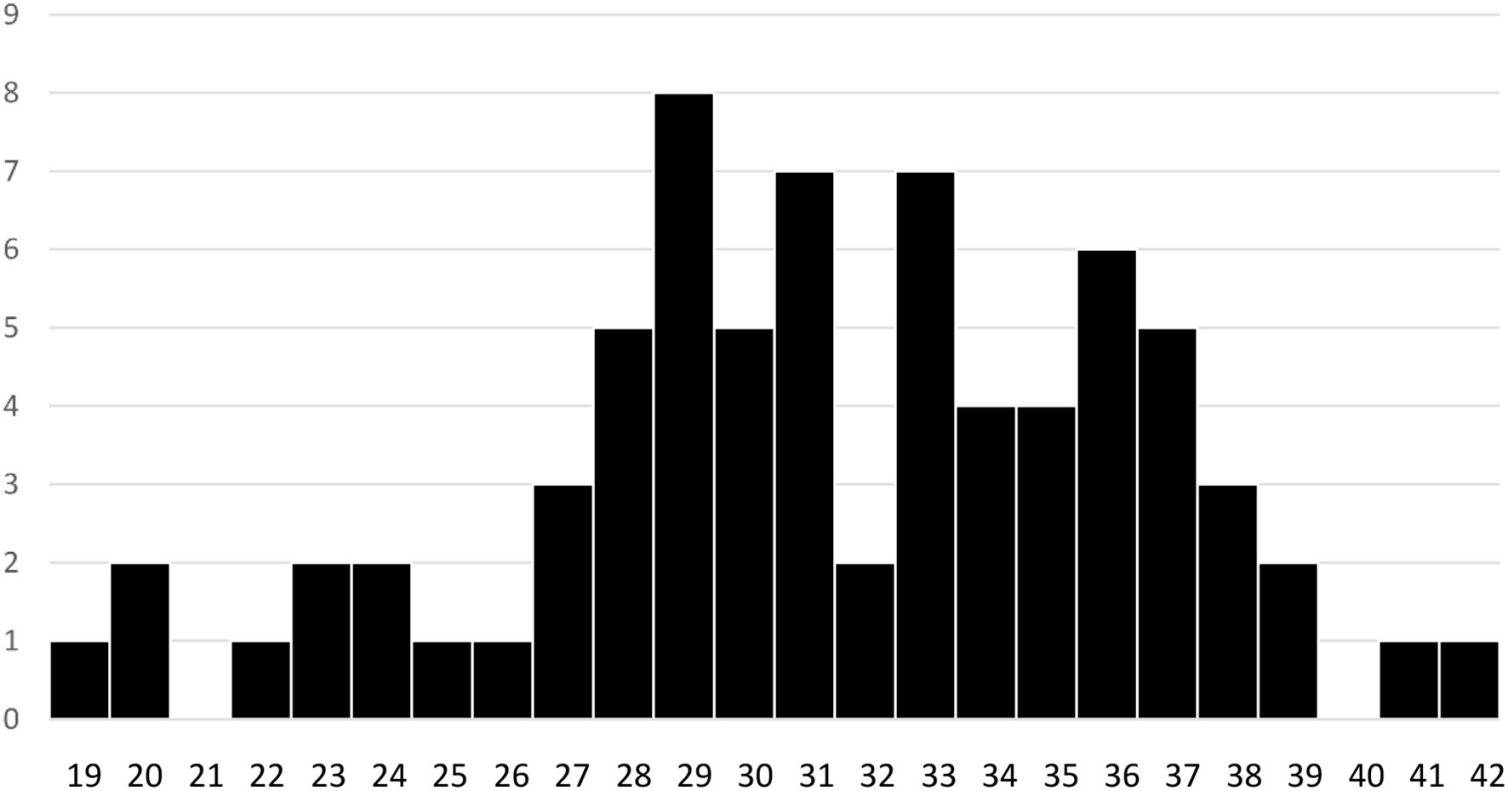
Ages of participants considering freebirth.

The youngest woman was 19, and the oldest was 41. The man was 42, but his partner's age is unknown. The average age was 31.4 ± 5 years, and the spread of ages are shown in Figure 3. The same person who declined to indicate their ethnicity or sexuality, also declined to indicate their age.

In terms of geographic distribution, participants considering freebirth were not confined to any particular location in the UK. There is representation in England, Scotland, Wales and Northern Ireland (see Table 2). There is largely no clustering in any of the NHS healthcare trusts, with the exception of three cases in the Nottingham University Hospitals NHS Trust.

**Table 2:** Geographical distribution of participants considering freebirth.

**England**	**54**
North West	6
North East	1
Yorkshire and Humber	4
West Midlands	4
East Midlands	12
East	6
London	5
South East	11
South West	4
**Scotland**	**8**
Highlands and Islands	3
Mid East Scotland	2
South East Scotland	1
South West Scotland	2
**Wales**	**4**
South Wales	3
North Wales	1
**Northern Ireland**	**2**
**Other**	
Guernsey	2
Did not fill in	1

The demographic characteristics of those considering freebirth were similar to the demographic characteristics of the entire dataset, with the exception of sexual orientation. Bisexual, lesbian and pansexual respondents made up 4.2% of all survey respondents, but 13.9% of the respondents considering freebirth. Sexual minority women were therefore more likely than heterosexual participants to be considering freebirth. Contingency table testing was used to determine if this difference was statistically significant. Fisher's Exact test was applied to the data, comparing the number of LGBQ+ participants in the full dataset with the number of LGBQ+ participants in the subset who had considered freebirth. This test showed that there was a difference between the groups, with LGBQ+ people being more likely to have considered freebirth (*p* < 0.001).

Although we did not collect demographic data about the profession of either the pregnant person or their partner, several respondents mentioned it within their responses to the open questions. One woman was a senior medical professional, two others work clinically within the NHS, two are non-clinical birth workers, another's partner is a GP, and one's husband is a Registered General Nurse (RGN). It is interesting both that so many people with professional experience in either birth or healthcare were considering freebirth, and that they felt it was important to provide this information in their answers. For those with partners who are in current clinical practice, this also presents a challenge to the definition of freebirth as a birth “without health care professionals (HCPs) present (1).” We will consider this further in the discussion.

### Plans Before the Pandemic

Interestingly, only one person who answered the survey had been planning to freebirth before the pandemic. The other participants had a range of birth plans. Many had been intending to birth at home (60). In England and Wales, around 2% of babies are born at home each year, meaning that those who had planned a homebirth are over-represented in this cohort (18). A significant proportion of respondents had also been considering giving birth in either a freestanding birth center, or an alongside midwife-led unit (11), whilst two women had been intending to give birth on the labor ward, and one woman had been intending to have a planned cesarean birth. Many respondents described that they had flexible plans for birth:

“If pregnancy remains low risk to go to [named] Birthing Center. Is [sic] any complications developed to go to [named] Hospital.”

Although all of the participants had seriously considered freebirth or were currently considering it at the time they completed the survey, there were a mixture of current plans for birth. Only two women had given birth before the survey, and of these, one woman had had a freebirth, whilst the other had seriously considered freebirth, but in the end had been able to obtain the midwifery care that she had been told would not be available. She explained that although the homebirth service was officially withdrawn:

“when my husband rang whilst I was in labor, they initially said no one could come, but after my husband asked to speak to the head of Midwifery, they said they could send someone out to do “checks” prior to transferring in. In the end, though, the midwife turned up with all the gear be and was happy to stay. Birth was extremely straightforward and fast (30 min after midwife arrived).”

Of the other 70 respondents whose babies had not yet been born, some were definitely intending to freebirth, whilst others remained undecided in their plans, and one woman was clear that she had previously seriously considered freebirth but was currently intending to give birth in hospital. The majority of expectant parents considering freebirth during the pandemic experienced negative feelings. Positive feelings seemed to be more prevalent amongst participants who had made the decision to have a freebirth, whilst those who were still undecided did not seem to share these positive feelings. Once the decision to freebirth had been made, participants described a returning sense of safety and security: “I feel safe in my own home.”

### Qualitative Findings

This section will use the qualitative data to explore the two remaining themes relating to the birth care and support respondents considered, and the reasons that respondents considered freebirth.

#### Options Considered

Expectant parents in this study had a range of different first choices for birthplace, including homebirths, birth centers and MLUs, labor wards, and elective cesarean births. When expectant parents' plans for birth changed because of lockdown, a freebirth was not always their second choice for birth either. Some women's second preference was to give birth in a different NHS setting, which they had been informed was not available to them. These difficulties are shown by this participant, as she explains why her second choice of birthplace was not available to her, for reasons unconnected to Covid-19:

“I have been told that the home birth service has been pulled and I won't be eligible for a midwife unit led birth as my BMI was too high at booking in so I am now planning to freebirth.”

Thirteen women in the study had considered using an independent or private midwife. These are fully qualified midwives, who are registered with the Nursing and Midwifery Council in the same way as NHS midwives. Independent midwives are self-employed, whilst private midwives are employed by private companies. Four women had hired an independent midwife, at the time of the survey. However, more women commented that they were unable to hire an independent midwife. For most, this was because they could not “afford it,” whilst for others it was because the independent midwives had no availability. One woman had considered hiring an independent midwife before lockdown, but had spoken to their maternity services who had reassured her they would be supportive of a home vaginal birth after cesarean with the result that she decided not to hire an independent midwife.

Unfortunately, the local homebirth service had then been suspended, and the independent midwife no longer had any availability. The participant commented, “I feel the decision has been made too quickly without thorough troubleshooting.” In another case, a respondent recalled that the local NHS Trust had:

“[I]n their infinite wisdom decided to cancel indemnity for all independent midwives in the area….Combined with the cancellation of NHS home births, women in my area are left with few choices of any.”

This meant that independent midwives were not legally able to attend births at that time.

The majority of participants who were considering freebirth because of Covid-19 had considered at least one other option subsequent to the changes in their original birth plans. Freebirth was therefore not a first or second choice for the majority of participants who were considering it.

#### Reasons for Considering Freebirth

Given that freebirth was the first choice of only one participant and was not even the second choice for many people, understanding the reasons why participants were considering it is important for healthcare services. The reasons given by expectant parents were varied. As Table 3 shows, they can be divided into three overarching categories: a desire to avoid hospital, birth preferences, and practicalities.

**Table 3:** Reasons why participants were considering freebirth.

**Avoiding hospital**	**Birth preferences**	**Practicalities**
Traumatic last birth	Birth partner excluded	Lack of childcare
Fear of hospitals	Access to water	Previous fast labor
Last baby died in the hospital	Desire for certainty	Distance to hospital
Concerned about cascades of intervention		No access to suitable transport
Fear or experience of coercion		
Risks of contracting Covid-19		

These reasons were not mutually exclusive, and many participants expressed several reasons for considering freebirth. Some of the reasons were also connected, for example:

“I will have to go into hospital alone as my husband doesn't drive and will have to look after our eldest daughter; there is no one else who can take her and she's not allowed to visit either.”

This section will explore each of the three main themes for considering freebirth.

#### Avoiding Hospitals

Thirty-nine participants said they were considering freebirth partly or wholly because they wished to avoid going into the hospital to give birth. For some this was due to past experiences giving birth in hospitals. For others, the potential of catching Covid-19 whilst in hospital felt too much of a risk to take. Rather than hospitals being a place where they and their babies would be safe, they had become places of potential danger and contamination for some women.

Some participants feared what would happen if they went to hospital for this birth. Women described being afraid of being coerced into interventions they did not want if they were in hospital or treated badly in other ways. These fears were not unrealistic, as they were often based on their previous experiences of hospital births:

“Despite having quick births ‘easy’ births I have been treated awfully during labor and for that reason only feel I have had one positive birthing experience. I was hoping this birth would be healing….”

Other women's fears were based on their experiences of care during this pregnancy, where they felt that coercion and “bullying” had already happened to them. These fears were compounded by the idea that they might be in hospital without a partner “to advocate for me.”

Hospital policies around the admission of partners to the labor ward were felt to be coercive by some women. Two women explained that their hospitals were only allowing partners in when labor was established. They had been informed that this would be judged by cervical dilation. However, cervical dilation can only be established by a vaginal examination. Two women described that they intended to decline the offered vaginal examinations but were scared that doing so would mean their partners were not allowed into the labor ward. The very fact that the stated policy made a partner's presence conditional on the women accepting an intervention made them feel that coercion was openly advertised as being integral to choosing a hospital birth.

For women whose partners or children were in the high-risk groups, going into hospital meant not only a risk to their own health and their newborn baby's health. It also meant that they potentially became contaminated, and a danger to their families. The dual hospital risks of interventions and the risk of contracting the virus were interrelated:

“I fear the changes are going to lead to [more] unnecessary interventions. And an increased risk therefore of having to stay in hospital, increasing the chance that me, baby and my husband's will be exposed to the virus. My husband has a heart condition so I fear the worst.”

#### Birth Preferences

Most NHS Trusts adopted a policy of only allowing one birth partner into labor wards, MLUs and birth centers during established labor. This created fear in some women that they would not have a known person with them for some or all of their labor. As well as wanting partners to be present at the birth to advocate for them, women described needing their support. This was especially the case when the journey to this birth had been difficult:

“[M]y partner is a great support for me, we have gone through IVF and a miscarriage together and I couldn't imagine doing any of this without him….”

Some NHS Trusts adopted a policy that the sole birth partner had to be someone the woman lived with, ostensibly to reduce the potential for Covid-19 transmission to healthcare professionals (19). This caused specific problems for single mums, those whose partners needed to stay with older children, and those whose partners had jobs where the risk of being affected by Covid-19 was high:

“[What] if my husband becomes locked down at work (possibility as he is [a] prison officer, when it hits the prisons they plan on literally locking the gates—in or out)….”

Many of the women who were in this position had planned their support carefully. Until just a few weeks before the survey, they had expected to be able to have a birth partner who they did not live with support them during birth—usually a doula (a non-medical birth worker who provides emotional and practical support), though one participant had intended to have her mother as her birth partner. Some of these women had intended to give birth in hospital or in birth centers and MLUs, with the support of their non-resident birth partner. They were very aware that they suddenly faced the real possibility of giving birth with no-one they knew present to support them.

In some NHS Trusts, the rules about who could be present at a birth were extended to homebirths as well. This created an impossible situation for one participant who is a single parent:

“Home births so far are still going ahead in my trust, however I wouldn't be allowed my doula or my kids in the room. I have no childcare and no other birthing partner.”

This situation had forced her into considering a freebirth, despite the fact that a homebirth service was still available.

For three women, access to water as a form of pain relief was an essential part of their birth plan. One participant was clear that she would have considered a waterbirth on the labor ward, but the only room with a pool was reserved for women who were Covid-19 positive or Covid-19 symptomatic^1^.

The number of changes and the uncertainty over which services might be available were mentioned by three participants as a factor in their consideration of freebirth. Different NHS Trusts have made changes to the services available at varying times. Service changes impacted expectant parents' plans, as they made new choices depending on the services available. A participant who had changed her plans several times already in response to the withdrawal and reinstatement of birth support by her NHS Trust said she was now considering freebirth because she did “not want to change my birth plans [again].”

A sentiment which was repeated by many participants was the feeling that they had been left with no choices by their perinatal services, with 26 participants describing feeling trapped, and forced into decisions that they did not want to make. They characterized the choices that they had, due to a combination of personal circumstances and local Trust policies as being “no choice” or an “impossible choice.” There was a sense that the decision to freebirth was one which the NHS services were making for them: “I feel I am being backed into a free birth.”

#### Practicalities

Some expectant parents were considering freebirth because of practical reasons, which were often multifaceted. Lockdown restrictions, and elderly parents shielding had restricted the childcare options available for older children for some families. If the partner was the only person available to take care of the children, and the homebirth service had been withdrawn, that meant being without known support during birth. For those whose partner could not drive, or without access to a vehicle, simply getting to the hospital could be a logistical problem. This was especially the case if a homebirth service had been withdrawn and local birth centers were closed, or not available because the pregnant person was not “low risk.” In rural areas, some women were faced with a significant journey to the only available NHS support for birth: “hospital 45 miles away.”

Even with access to a car and a driver, this is a daunting journey to undertake in labor. Without that access, options were very restricted:

“We don't have a car, and the idea of taking a taxi in mid labor, during a virus outbreak, was unthinkable.”

Concern about the distance that might need to be traveled whilst in labor was compounded by previous birth history when women had had fast labors. The woman who lived 45 miles from the hospital said one of her main reasons for considering freebirth was that:

“My last baby was born in less than an hour and a half so I'm worried I wouldn't make it to the hospital.”

In total, eight participants mentioned that a previous history of precipitous labor was a factor in their consideration of freebirth. All of these women had previously planned a homebirth, or a birth in a birth center with close proximity to their home. They did not perceive that they were making a choice between giving birth in a hospital and freebirthing, but rather between freebirthing and “End[ing] up having an accidental unassisted birth.”

## Discussion

This is the first large scale study to capture the demographics of people contemplating freebirth within the UK. It is also the first study to identify LGBTQ+ people considering freebirth. Importantly, freebirth was contemplated by people throughout the UK suggesting that this decision was not motivated by the actions of a few restrictive NHS trusts, but rather that the issue was far more widespread. Furthermore, as far as we are aware, this is the first freebirth study to capture data from all four countries of the UK.

### Characteristics of Those Who Considered Freebirth

Notably, this is also the first time that a UK study has shown that NHS health care professionals have contemplated stepping outside of the NHS maternity system in order to freebirth their babies. As no respondent mentioned other, unconnected professions, it appears that respondents may have been justifying their choice to consider freebirth by constituting themselves or their partners as experts. This also raises as yet unanswered questions about NHS staff perception of safety in relation to the service they and their colleagues provide. It also offers a challenge to the definition of freebirth. If either the person who is giving birth or their partner is currently in clinical practice, can the birth be said to be “without health care professionals (HCPs) present (1)?”

We note that participants within our survey have specifically used the term “freebirth,” alongside responses that indicate that they or their partners are healthcare professionals, and we believe it is important that their terminology about their birth choices is respected. The term was also used by most participants in the survey without healthcare training or partners. Using the term “freebirth” is an active, linguistic choice indicating an awareness of it as a social phenomenon. Moreover, those that indicate they or their partners are healthcare professionals, will likely have awareness of the stigma of freebirthing. We do not propose to offer an alternative definition of freebirth here, but instead highlight this as an issue for consideration should further research into health care professionals stepping outside the NHS maternity system be undertaken.

It is well-established that pregnant lesbian and bisexual women face routine heteronormativity, invisibility and invalidation in their encounters with perinatal care (20). Research also shows that LGBTQ+ people may experience fear and discomfort when accessing healthcare services; that fear being based on frequent accounts of other LGBTQ+ people being denied access to healthcare services or discriminated against when they disclose their gender or sexual orientation (21). A small amount of research shows that lesbian and bisexual women may even face hidden physical assault in perinatal care, such as deliberately rough vaginal examinations (22). We do not know whether this community experience of poor care was a factor in LGBTQ+ people choosing to freebirth in this study, but fear of poor care is a motivating factor that has been identified in other freebirth research (see for example 4). Other studies have not identified LGBTQ+ people choosing to freebirth before, and research into LGBTQ+ birth choices have not identified freebirth as a possible decision. Further research in this area is needed to understand whether LGBTQ+ people considering freebirth come from similar or different motivations than cis-heterosexual people.

### The Importance of Choice

Anyone can legally choose to give birth at home, regardless of whether this would be medically recommended. This is a well-established right, which has been confirmed under European law (23). Birth centers and MLUs can have their own policies about who is allowed to give birth there. NHS England says that the place of birth should be decided by the person who is pregnant:

“Women should be able to make decisions about the support they need during birth and where they would prefer to give birth, whether this is at home, in a midwifery unit or in an obstetric unit [(24), p. 9].”

However, in many NHS Trusts there is a policy that only women deemed “low risk” can give birth in birth centers or MLUs. The National Institute of Clinical Excellence (NICE) suggests that only around 45% of pregnancies are considered “low risk” (25). This means that when a homebirth service is withdrawn, many people may only be able to give birth in the hospital labor ward if they want NHS healthcare professionals' support during the birth, even if the birth center or MLU remain open.

Research is shortly due to be published that shows which perinatal choices different NHS Trusts were able to maintain, and which they decided it was necessary to remove. These results are welcome, and important for future emergency planning of perinatal services. As these findings show, removal of choice leads to pregnant people who would rather have an attended birth considering freebirth. However, the stories above also show that personal circumstances can mean that the maintenance of choice in birth is not as simple as which of the four places of birth are open. If a birth center is kept open when a homebirth services is closed but is only available to those who are “low risk”, it does not provide choice for most people. If a homebirth service is still running, but children and those from other households are not allowed in the room, it is not a service that can be used by single parents. If a single birth supporter is allowed, but they have to be from the same household, single pregnant women and people face giving birth without support from someone they know. As can be seen in the responses to this survey, it can be the most vulnerable people who are affected by service disruption the most, and who then feel they are left with no choice but to consider freebirth. Choices which are seen as clinically minor choices (such as access to a birth pool on a labor ward) may be of great importance to pregnant people when making decisions about birth. It is therefore important that quantitative research into the choices that NHS Trusts were able to maintain is nuanced to service users' choices and takes into account the ways different personal circumstances may interact with perinatal service availability or restriction.

Although this study of freebirth took place during the Covid-19 pandemic it becomes apparent that pregnant people's motivations reflect those noted by previous scholars. Concern about the safety of hospitals, the reduction of homebirth options, the practicalities of attending hospital and previous birth trauma were all important motivations in this cohort. This demonstrates that the Covid-19 pandemic has placed a spotlight on existing problems in maternity care. Data from this study is clear: when pregnant people are presented with a maternity service they deem unsafe or does not align with their needs, desires or world view, they may step outside of that system. If service providers wish to ensure people have access to perinatal maternity care, they must provide a service that is acceptable to those who are using it.

This study has also exposed how some pregnant people considered maternity policies as coercive. A fear of being coerced into unwanted medical interventions raises serious issues regarding the under-researched area of informed consent and refusal in NHS maternity care. It must be ensured that policies do not inadvertently subvert informed consent as this could result in those giving birth submitting to interventions they may otherwise have refused. As already highlighted above, a desire to avoid such policies was a motivating factor for some people in this cohort.

Freebirth as a subject of academic research has only begun to be studied relatively recently, and the literature pertaining to it is small. The available literature suggests that it is a decision pregnant women make for a variety of reasons, including previous traumatic births (6), a lack of support for birth choices (4) and a belief in the inherent safety of undisturbed physiological birth (3). This research suggests that a global pandemic represents a new factor in such decisions.

### Risk

Although the concept of risk typically dominates discussion on pregnancy and childbirth, the Covid-19 pandemic appears to have challenged people's views on where and how it is safest to give birth. Hospitals are generally assumed to be places of safety, however for women who have experienced a traumatic birth, or who are worried about iatrogenic harm in birth, hospitals may feel unsafe (26). During the pandemic, hospitals have become viewed by many people as risky places to be avoided, where the risk of Covid-19 transmission is high (27), and this fear was expressed by participants in this research too. Conversely, freebirth may be assumed to be a risky choice, and those who choose to freebirth are sometimes accused of making choices for their own benefit whilst disregarding the safety of their baby. Participants in this survey who were considering freebirth because they wished to avoid hospitals were clear that they were putting safety first. The vast majority of people within this study had not considered freebirth before the pandemic, but Covid-19, birthing restrictions and rapidly changing policies created competing risks that meant freebirth became an acceptable option. This indicates the complexity of people's decision-making and demonstrates how people's understanding of risks associated with place and manner of birth are not limited to what may be deemed a medical calculation of physical risks.

### Strengths and Limitations

This project provided a brief snapshot into the thoughts, feelings, and decisions of expectant parents in the first weeks of the Covid-19 lockdown in the UK. There is an immediacy to these qualitative responses that can provide researchers, policy makers, and practitioners with an insight into lived experiences. The numbers considering freebirth, and the reasons that they were considering this could usefully inform reorganization and prioritization of perinatal services in the event of future lockdowns.

The research was intended to capture experiences from a wide range of expectant parents, and freebirth was not a specific area of investigation within the research. Capturing data from so many people considering freebirth was unexpected. Data capturing the number of freebirths are not routinely collected in the UK, apart from in London, where this information can be volunteered by parents (28). Through Freedom of Information requests to Health Boards some data is available for Wales, but here the numbers also include cases where a baby was born before the arrival of a midwife at home, or the parent at a hospital, MLU or birth center (28). We cannot therefore know if the 72 participants considering freebirth in this research represents a greater than usual proportion. Additionally, as most people who answered the survey had not yet given birth, we can only state how many people *considered* freebirth, and cannot know the numbers of those who eventually decided to do so. A limitation of this real-time survey tool is that the resultant dataset is a convenience sample which may be biased toward those that feel most strongly about their pregnancy experiences. It could therefore be that those expectant parents who were considering freebirth were more likely to complete this questionnaire than parents who felt more sanguine about the available NHS birth choices.

### Future Research Directions

Further research into perinatal experiences during the Covid-19 pandemic has already been planned and partially conducted both within the UK and internationally. The results of other studies will fill some of the research gaps within this work. The opportunity to compare these findings on an international level would also create a more nuanced understanding of the circumstances that affect the consideration of freebirth during a pandemic.

As mentioned above, it is not currently known how many participants considering freebirth went on to have a freebirth within this study. Follow-up research to determine the actual circumstances of birth, and participants' satisfaction with their decisions could provide useful information, as no freebirth research to date has focused on consideration of freebirth.

This research suggests for the first time that specific groups of people may be more likely to have considered freebirth during the Covid-19 pandemic. Further research with LGBTQ+ people and HCPs would be useful to establish whether these groups are more likely to consider freebirth outside of a pandemic, and to understand the reasons why this might be.

## Data Availability Statement

The data will be made available without undue reservation through the UK Data Service, in accordance with ESRC protocols.

## Ethics Statement

The studies involving human participants were reviewed and approved by King's College London, BDM Research Ethics Subcommittee. The patients/participants provided their written informed consent to participate in this study.

## Author Contributions

MG: conceptualization, methodology, formal analysis, writing—original draft, and writing—review and editing. SP-G: formal analysis, writing—original draft, and writing—review and editing. GM: writing—original draft and writing—review and editing. All authors contributed to the article and approved the submitted version.

## Conflict of Interest

The authors declare that the research was conducted in the absence of any commercial or financial relationships that could be construed as a potential conflict of interest.

## Footnotes

^1^From the larger survey, the reserving of pool rooms for women with Covid-19 appears to be a common practice, even though women with Covid-19 are not supported in having a waterbirth in most NHS Trusts.

## References

[1] McKenzie G Robert G Montgomery E. Exploring the conceptualization and study of freebirthing as a historical and social phenomenon: a meta-narrative review of diverse research traditions. Med Human. (2020) 46:512–24. 10.1136/medhum-2019-011786 PMC7786152 32361690

[2] Spencer-Freeze R. Born Free: Unassisted Childbirth in North America. PhD Thesis University of Iowa (2008). 10.17077/etd.gqjehps0

[3] Feeley C Thomson G. Why do some women choose to freebirth in the UK? An interpretative phenomenological study. BMC Pregn Childbirth. (2016) 16:1–12. 10.1186/s12884-016-0847-6 27000100 PMC4802706

[4] O'Boyle C. Deliberately unassisted birth in ireland: understanding choice in Irish maternity services. Br J Midwifery. (2016) 24:181–7. 10.12968/bjom.2016.24.3.181

[5] Cameron HJ. Expert on Her Own Body: Contested Framings of Risk and Expertise in Discourses on Unassisted Childbirth: Master's Thesis Lakehead University. (2012). Available online at: https://knowledgecommons.lakeheadu.ca/handle/2453/526 (accessed February 9, 2021).

[6] Jackson M Dahlen H Schmeid V. Birthing outside the system: Perspectives of risk amongst Australian women who have high risk homebirths. Midwifery. (2012) 28:561–7. 10.1016/j.midw.2011.11.002 22300611

[7] Henriksen L Nordström M Nordheim I Lundgren I Blix E. Norwegian women's motivations and preparations for freebirth—A qualitative study. Sex Reprod Healthcare. (2020) 25:100511. 10.1016/j.srhc.2020.100511 32283477

[8] Holten L de Miranda E. Women's motivations for having Unassisted Childbirth or high-risk homebirth: an exploration of the literature on 'Birthing outside the system. Midwifery. (2016) 38:55–62. 10.1016/j.midw.2016.03.010 27055760

[9] Kornelsen J Stefan G. The reality of resistance: the experiences of rural parturient women. J Midwifery Women's Health. (2006) 51:260–5. 10.1016/j.jmwh.2006.02.010 16814220

[10] RCOG. Coronavirus (COVID-19) Infection and Pregnancy. (2020). Retrieved from: www.rcog.org.uk/en/guidelines-research-services/guidelines/coronavirus-pregnancy (accessed March 30, 2020).

[11] Birthplace. (2011) Retrieved from: https://www.npeu.ox.ac.uk/birthplace (accessed March 30, 2020).

[12] Walsh D Spiby H McCourt C Grigg C Coleby D Bishop S. Factors influencing the utilization of free-standing and alongside midwifery units in England: a qualitative research study BMJ Open. (2020) 10:e033895. 10.1136/bmjopen-2019-033895 PMC7045002 32071182

[13] AIMS. Coronavirus and Your Maternity Care. (2020). Retrieved from: https://www.aims.org.uk/information/item/coronavirus (accessed September 01, 2020).

[14] Birthrights. Birthrights Calls for Easing of Visiting Restrictions in Maternity Services. (2020). Retrieved from: https://www.birthrights.org.uk/2020/08/20/birthrights-calls-for-easing-of-visiting-restrictions-in-maternity-services (accessed September 01, 2020).

[15] RCM. Clinical Briefing Sheet: ‘freebirth' or ‘Unassisted Childbirth' During the COVID-19 Pandemic. (2020). Retrieved from: https://www.rcm.org.uk/media/3923/freebirth_draft_30-april-v2.pdf (accessed August 25, 2020).

[16] Braun V Clarke V. Using thematic analysis in psychology. Qualitative Res Psychol. (2006) 3:2:77–101. 10.1191/1478088706qp063oa

[17] Boyatzis RE. Transforming Qualitative Information: Thematic Analysis and Code Development. Thousand Oaks, CA, London: Sage Publications (1998).

[18] Office for National Statistics. Birth Characteristics In England And Wales: 2019. (2020) Retrieved from: https://www.ons.gov.uk/peoplepopulationandcommunity/birthsdeathsandmarriages/livebirths/bulletins/birthcharacteristicsinenglandandwales/2019#place-of-birth (accessed December 18, 2020).

[19] RCM. Coronavirus Q A. (2020). Retrieved from: https://www.rcm.org.uk/coronavirus-qa/ (accessed April 30, 2020).

[20] Rondahl G Bruhner E Lindhe J. Heteronormative communication with lesbian families in antenatal care, childbirth and postnatal care. J Adv Nurs.(2009) 65:2337–44. 10.1111/j.1365-2648.2009.05092.x 19737324

[21] Light AD Obedin-Maliver J Sevelius JM Kerns JL. Transgender men who experienced pregnancy after female-to-male gender transitioning. Obstetr Gynecol. (2014) 124:1120–7. 10.1097/AOG.0000000000000540 25415163

[22] Spidsberg BD. Vulnerable and strong – lesbian women encountering maternity care. J Adv Nurs. (2007) 60:478–86. 10.1111/j.1365-2648.2007.04439.x 17973711

[23] Ternovszky v. Hungary. Application no. 67545/09, European Court of Human Rights. (2011). 21644439

[24] National Maternity Review. Better Births: Improving outcomes of maternity services in England - A Five Year Forward View for Maternity Care. (2016). Available online at: https://www.england.nhs.uk/wp-content/uploads/2016/02/national-maternity-review-report.pdf?PDFPATHWAY=PDF (accessed February 9, 2021).

[25] NICE. Midwife-Led Units Safest for Straightforward Births. (2014). Retrieved from: https://www.nice.org.uk/news/article/midwife-led-units-safest-for-straightforward-births (accessed August 18, 2020).

[26] Lyndon A Malana J Hedli LC Sherman J Lee HC. Thematic analysis of women's perspectives on the meaning of safety during hospital-based birth. J Obst Gynecol Neonatal Nurs.(2018) 47:324–32. 10.1016/j.jogn.2018.02.008 29551397 PMC5938121

[27] The Health Foundation. People Are Avoiding Hospital Because They Are Nervous of Catching COVID-19. (2020). Retrieved from: https://www.health.org.uk/news-and-comment/news/people-are-avoiding-hospital-because-they-are-nervous-of-catching-covid (accessed September 01, 2020).

[28] Bryan N. Freebirth data 'should be collected across (2018). UK' BBC News Retrieved from: https://www.bbc.co.uk/news/uk-wales-south-east-wales-42706652 (accessed September 01, 2020).

