# Supplementary material for: Between a Rock and a Hard Place: Considering “Freebirth” During Covid-19
Source: Front Glob Womens Health. 2021 Feb 18;2:603744. doi: 10.3389/fgwh.2021.603744 (PMC8594025; doi:10.3389/fgwh.2021.603744)
Supplement: Supplementary file 1 [file Table_1.DOCX]

**Annex 1 – codebook for themes identified**

| **Theme** | **Subtheme** | **Frequency** | **Description** | **Example** |
| --- | --- | --- | --- | --- |
| Planned place of birth |  | 72 | States intention to birth in a specific place or in a specific way | Plan for obstetric delivery suite |
| Non-NHS support available/considered | Doula | 22 | Impact of Covid-19 on choice to use/not use a doula | Worried as the doula was to provide support after previous bad experiences |
|  | Independent midwife (IM) | 13 | Considers whether using an independent midwife to secure birth choices is possible | I am afraid that I am being pushed to put myself in financial hardship to avoid having to end up on a labour ward or if we cannot secure an IM |
| Reasons for considering freebirth | Avoid hospital | 39 | Wants to avoid going into hospital | I am considering a Freebirth as I really want to avoid the hospital |
|  | Previous traumatic birth | 10 | States previous traumatic birth is a reason for considering freebirth | I desperately don't want to birth in hospital after my previous traumatic birth |
|  | Coercion | 12 | Is considering freebirth as concerned about coercion | I’m afraid if I end up in a labour ward I will be alone and potentially coerced into a cascade of interventions. |
|  | Birth partner potentially excluded | 24 | Concerned that their birth partner may be excluded from some or all of the time that they would want support | fear of my husband being unable to support me at the birth (if he was symptomatic) |
|  | Uncertainty | 3 | Considering freebirth because of the uncertainty of the NHS services that will be available when they give birth | it’s been extremely disorganised with little to no information being given out about changes |
|  | Access to water | 3 | Considering freebirth because they wish to access water during birth | some places are trying to restrict access to pools, even at home. |
|  | Childcare | 11 | Considering freebirth because of childcare difficulties | I now have a child care issue as my 3year old could have stayed with us at home. Both sets of parents are in the shielding category and we live over 30 mins away from other family |
|  | Distance/access to transport | 4 | States the distance to hospital and/or lack of appropriate transport as a reason for considering freebirth | We don't have a car, and the idea of taking a taxi in mid labour, during a virus outbreak, was unthinkable. |
|  | Timing | 7 | Mentions previous precipitous labour as a reason for considering freebirth | My last baby was born in less than an hour and a half so I'm worried I wouldn't make it to the hospital |
| Feelings | About Covid-19 | 24 | Mentions any feelings about the virus | Considering free birth rather than having to go into an understaffed hospital full of dangerously infectious patients |
|  | Negative | 87 | Negative feelings in relation to pregnancy, birth, or changes to plans | Like I’ve had my entire pregnancy shat on |
|  | Positive | 16 | Positive feelings in relation to pregnancy, birth, or changes to plans | Liberated. I have been able to reject NHS care without being questioned as much and I think my birth will be a lot less stressful now |
|  | About freebirth | 25 | Describes any feeling in relation to freebirth | W e are now considering to freebirth, which would have never crossed my mind before! |
|  | Lack of choice | 26 | Mentions a lack of choices | Choice has been removed and so has an element of safety. |
|  | Understanding | 6 | Mentions understanding the changes that have been put in place by the NHS | Obvious upset but there is an element of understanding there |
|  | Regretting pregnancy | 6 | Mentions regretting their pregnancy | I wish I wasn’t pregnant |
